# Rollout Strategy to Implement Interoperable Traceability in the Seafood Industry

**DOI:** 10.1111/1750-3841.13744

**Published:** 2017-08-21

**Authors:** Martin Gooch, Benjamin Dent, Gilbert Sylvia, Christopher Cusack

**Affiliations:** ^1^ Value Chain Management Intl. Inc. 1155 North Service Rd. West, Suite 11 Oakville ON L6M 3E3 Canada; ^2^ Coastal Oregon Marine Experiment Station, Oregon State Univ. Hatfield Marine Science Center 2030 Marine Science Drive Newport OR 97365 U.S.A; ^3^ Yaquina Resources Group Corvallis OR 97333 U.S.A

**Keywords:** enterprise engineering, interoperability, seafood, strategy, technology architecture, traceability

## Abstract

Verifying the accuracy and rigor of data exchanged within and between businesses for the purposes of traceability rests on the existence of effective and efficient interoperable information systems that meet users’ needs. Interoperability, particularly given the complexities intrinsic to the seafood industry, requires that the systems used by businesses operating along the supply chain share a common technology architecture that is robust, resilient, and evolves as industry needs change. Technology architectures are developed through engaging industry stakeholders in understanding why an architecture is required, the benefits provided to the industry and individual businesses and supply chains, and how the architecture will translate into practical results. This article begins by reiterating the benefits that the global seafood industry can capture by implementing interoperable chain‐length traceability and the reason for basing the architecture on a peer‐to‐peer networked database concept versus more traditional centralized or linear approaches. A summary of capabilities that already exist within the seafood industry that the proposed architecture uses is discussed; and a strategy for implementing the architecture is presented. The 6‐step strategy is presented in the form of a critical path.

## Introduction

The preceding articles detailed why the effective and efficient sharing of data and information is critical to businesses operating along the supply chain possessing the ability to reduce costs and increase revenue from possessing the ability to continually adapt to changing circumstances and market demands. The articles also described why effective and efficient sharing of data and information is vital to enabling businesses and other industry stakeholders (including government agencies and nongovernment organizations [NGOs]) to mitigate environmental, ecological, social, and economic risks. The accuracy and rigor of data exchanged within and between businesses and other stakeholders rests on access to effective interoperable information systems. The ability to establish effective interoperability necessitates that systems used by businesses operating along the value chain share a common technology architecture (blueprint). A technology architecture describes a collection of interrelated specifications, standards, and practices for hardware, software, and communications interfaces which, together with core services, operate in service of a common goal. In this case, the service being enabled through the implementation strategy described in this article is interoperable chain‐length traceability across the global seafood industry.

The technology architecture described herein is a distributed peer‐to‐peer (P2P) networked database architecture. Compared to alternative architecture designs, having no central database provides a number of advantages. These advantages include scalability and robustness, which is crucially important for enabling users to effectively and efficiently use complex technologies in a challenging business environment. The technology architecture rollout strategy described in this document reflects the perspective that architectures are developed and implemented by engaging industry stakeholders in a purposeful dialogue on why the architecture is required, the benefits and opportunities its implementation offers industry, along with components and specifications required to transform a conceptual design into an implementable solution that meets the needs of industry and other stakeholders. This article incorporates findings from a literature review of lessons learned from both successful and unsuccessful attempts to implement complex information systems and technology solutions for traceability or other purposes.

We begin by highlighting prior research that has influenced the plan for rolling out the technology architecture, and then discuss the implementation process at length.

## Key Factors Influencing Rollout Strategy

Researchers from a range of industries (including food, manufacturing, and service) have identified the extent to which the competitiveness of businesses no longer comes primarily from transforming one product into another (Porter and Millar 1985; Gooch and Sterling 2013). Increasingly, competitiveness arises in using information technology to gather, select, organize, synthesize, and distribute data (Rayport and Sviokla 1994, 1995, 1996; Pine and others 1995; Prahalad and Hamel 1996) to produce information that enables the product transformation processes that occur along the supply chain to be continually improved in ways that would not otherwise be possible (Sterling and others 2015; Lewis and Boyle 2017). Traceability enables this to be achieved by providing a direct link between products flowing along a physical supply chain and information flowing along a virtual chain of data (Rayport and Sviokla 1995; Gooch and Sterling 2013; Sterling and others 2015).

Hardt and others (2017) are among those who have identified that the primary challenges facing the introduction of the information technology and a common technology architecture for enabling interoperable traceability are not technical in nature. The primary challenges to implementing interoperable traceability in seafood are cultural and attitudinal. Resistance to change, including the adoption of new technologies and approaches, is particularly evident in industries in which stakeholder relationships are typified by a sense of adversity (EFFP 2004; Taylor 2006; Gooch 2012). A sense of adversity heightens individuals’ fears that: (1) change will be thrust upon them by more powerful partners, with no benefit to themselves; and (2) change will potentially render them irrelevant or harm their career prospects. The seafood industry is typified by adversarial relationships between businesses that range enormously in size and power (Sterling and others 2015; Hardt and others 2017). Countering these fears, resulting in individuals’ acceptance to adopt changes in practice or technologies, requires motivating individuals to emotionally connect with a clear reason for why they should adopt what is being proposed. Without emotional connection, evidence regarding how and when to implement change becomes null and void (Wlodkowski 2008; Zull 2011; Gooch 2012).

Achieving the necessary technology capabilities is less of a barrier to change. Although the seafood industry is inherently complex, which presents challenges from design and implementation standpoints, the technology required to enable interoperable seafood traceability already exists (Bhatt and Gooch 2017; Bhatt and others 2017; Lewis and Boyle 2017). The support services required to enable the implementation of chain‐length interoperable traceability also exist (Bhatt and others 2017; Lewis and Boyle 2017). Countering attitudinal and cultural barriers that negatively impact the seafood industry's adoption of interoperable traceability requires that the rollout strategy engage industry to emotionally connect with why the solutions proposed are important and beneficial to individuals regardless of their place in the chain and the size or power of their business. Recognized industry leaders play an important role in establishing an emotional connection between individuals and a topic (Sterling and others 2015), especially in situations where a strategy is relatively new or untested across a wider industry (Schmitz Whipple and others 1999; Gooch 2012). As identified by Bhatt and others (2017), establishing a strong and effective governance model is an important preemptive step for encouraging the commitment of industry leaders to a new strategy, and to produce technology solutions that matter to industry.

Other system‐wide issues stemming in part from the extent to which the seafood industry does not emotionally connect with interoperable traceability (and traceability *per se*) include a lack of knowledge and informed perspectives towards the need, purpose and benefits of traceability from competitiveness and profitability perspectives (Sterling and others 2015; Bhatt and others 2016). Lack of knowledge towards a topic leads individuals to view alternatives from a dualism versus duality perspective (Ison and Russell 2000; Gooch 2012). Dualism is when individuals perceive options to be black or white (all or nothing). Duality is when individuals perceive many options (levels of gray) existing simultaneously. Individuals possessing a duality perspective are more likely to proactively embrace change.

The rollout strategy therefore needs to incorporate an effective means of motivating individuals to want to learn about traceability and how they can benefit from its implementation. Ways to achieve this include demonstrating the opportunities that will be missed and disadvantages experienced by not implementing traceability. The strategy's design and implementation also need to acknowledge that the amount and type of data or information shared by businesses will differ according to:

1. in which of the 4 types of seafood supply/value chains identified by Sterling and others (2015)—fragmented, cooperative, coordinated, and collaborative—in which they operate, and
2. where they are located along the chain (Lewis and Boyle 2017).

Proving the architecture's inherent flexibility for enabling businesses to benefit from traceability in different ways is therefore important to the strategy's success. This can be achieved through creating and actively sharing business case studies, such as those utilized by the produce traceability initiative (PTI). Case studies, communicated through a variety of avenues and industry leaders, have assisted PTI in quite rapidly achieving success. The value of creating business cases and actively communicating with industry through a variety of channels was also evident in other industries described in the article addressing lessons learned from other industries (Bhatt and others 2017) appearing earlier in this Supplement.

## Overview

The rollout strategy presents a critical path roadmap for transforming the strawmodel of the technical architecture detailed in the report, entitled “Specifications to Implement a Technology Architecture for Enabling Interoperable Food Traceability,” (Bhatt and Gooch 2017) for enabling chain‐length interoperable traceability in seafood, from concept to practice. The term “critical path” conveys that activities are presented in the order in which they must occur to successfully complete a project, with each activity needing to be completed prior to the subsequent activity commencing.

**Figure 1:**
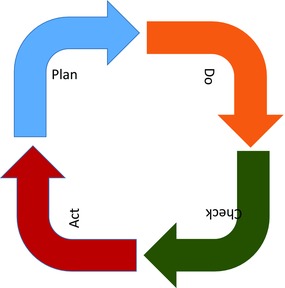
Plan do check act cycle.

The rollout strategy reflects the widely recognized and proven Plan→ Do → Check → Act (PDCA) concept for successfully implementing processes and technologies. Shown in Figure 1, the PDCA approach is a disciplined iterative framework that guides the design and testing of technologies and processes used to produce commercial value from their implementation, the impact of which is evaluated in a controlled environment before more ambitious efforts are undertaken.

The strategy therefore encompasses a series of preparation steps and strategic pilots that include stakeholders from developed and developing nations. The architecture will enable the robust operation of a scalable distributed P2P networked database. The rollout process will engage stakeholders in establishing the technical capabilities and motivation required to implement interoperable electronic systems in a highly complex industry. Although the initial efforts will primarily focus on North America, the resulting systems could be applied in multiple nations simultaneously. The rollout strategy constitutes a critical path to the architecture's implementation.

**Figure 2:**
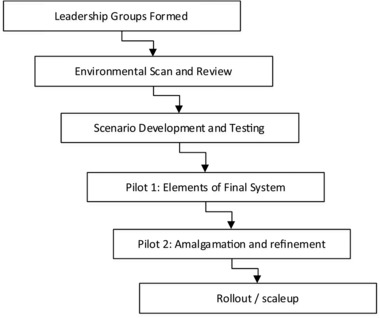
Rollout strategy overview.

To optimize the value offered by the resulting technology solution(s) to the seafood industry, the proposed strategy (shown in Figure 2) has 6 distinct phases. The importance of each phase to successful implementation of the technology architecture is briefly discussed prior to subsequent sections of the report describing in detail the activities that will occur during each phase of the rollout strategy. To ensure stakeholder's confidence in the system developed, each phase would end with a review to ensure that all objectives have been completed satisfactorily prior to moving forward to the next phase.

The need for a stepped approach is discussed by Thakur and Hurburgh (2009), who developed a framework for implementing a traceability system in the bulk grain supply chain. They discuss 3 phases to the implementation plan:

(1) First usage requirements (type of information, and so forth) are defined.
(2) A model is developed for implementing internal traceability for a single actor in the chain.
(3) A model is developed for information exchange between supply chain actors.

Feng and others (2013) also discuss designing and implementing a traceability system for the cattle industry in China following a stepped approach. After key traceability information was identified using a survey, a conceptual model was proposed. The system was then implemented using a few test enterprises.

The strategy's design was guided by a literature review to ascertain previous experience in developing and implementing technology architectures—particularly those suited to enabling traceability. Review findings were incorporated into this paper. Specific insights sought during the review included how to avoid building a hugely expensive “white elephant” that is either too impractical or too cost prohibitive to use, and therefore unsustainable. In manufacturing, individual components of a car can be tested, a prototype or scale model built, providing the company a good idea that the end product will function as expected. Companies can also build on their previous experience constructing similar products. For example, the evolution of passenger planes has been continuous, with each plane building on lessons learned from the previous model. Inherent complexities associated with IT and global information systems, however, prevent scale models from being built. It is not the number of users that matters most, it is functions and capabilities—which must be fully developed before a system can be tested in practice.

**Figure 3:**
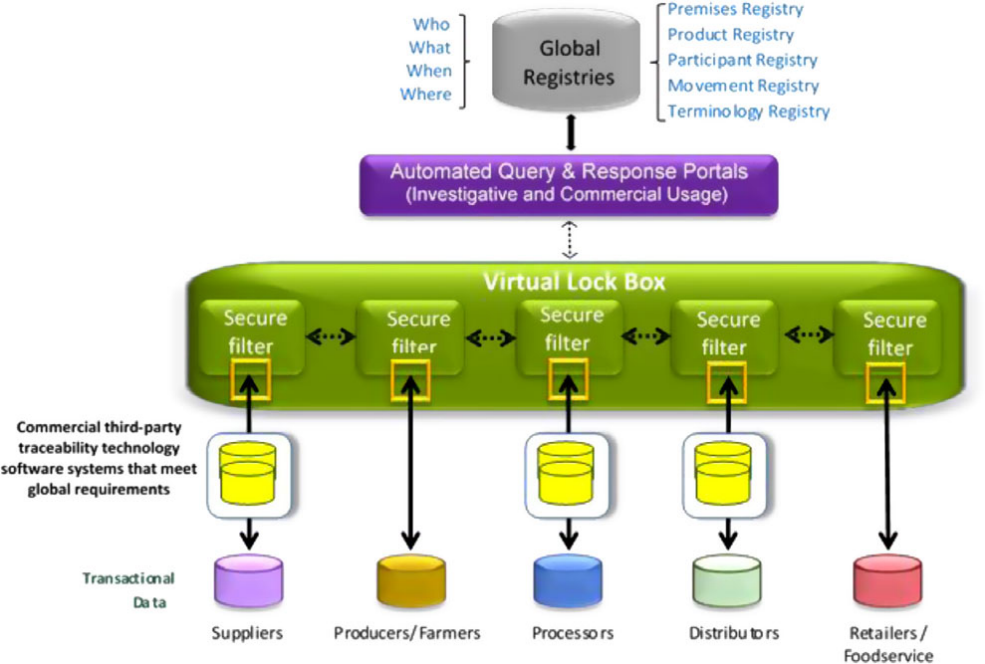
Interoperable traceability architecture (Bhatt and Gooch 2017).

The rollout strategy commences by briefly summarizing each of the rollout strategy's 6 phases presented in Figure 2. Activities that must occur during each phase of the rollout strategy to enable the implementation of the conceptual technology architecture shown in Figure 3 are subsequently described in detail.

This design addresses common data ownership and security concerns pertaining to interoperable traceability. A distributed system enables industry stakeholders and government agencies to maintain control of their data, which resides within their own internal database systems. The virtual lock box feature means that data are shared as needed. The technology architecture and its operation are detailed in the document entitled “Specifications to Implement a Technology Architecture for Enabling Interoperable Food Traceability” (Bhatt and Gooch 2017).

### Phase 1: Leadership groups formed

As described in prior articles, establishing a strong effective governance process plays a critical role in establishing industry support and commitment for the technology solutions and processes required to enable the implementation of sustainable interoperable traceability (Bhatt and others 2017; Lewis and Boyle 2017). Formation of the governance structure that will oversee the rollout initiative would be established during the process of finalizing the strategy, which will be achieved through communicating to industry the work achieved to date and capturing the resulting feedback.

### Phase 2: Environmental scan and review

The 2nd phase of the rollout strategy will provide an important foundation for the 4 subsequent steps. This phase will enable complementary capabilities to be identified, technology or process gaps to be quantified, and committed parties brought together in a coordinated fashion. Phase 2 will achieve this by mapping out what is presently occurring globally to determine the extent to which individual businesses are ahead of the curve in enabling interoperability. Phase 2 will also identify lessons learned from prior developments of large and complex IT systems, including the issues that led to previous IT initiatives not meeting their objectives or failing completely.

The insights and knowledge produced during Phase 2 will guide the creation of a framework for transforming distributed P2P and related capabilities developed from a variety of environments into technology and process solutions suited to achieving interoperability in seafood. Reflective of considerations required to complete real options and cost benefit analyses, this framework will provide a key input required to accomplish Phase 2 of the rollout strategy.

### Phase 3: Scenario development and testing

The 3rd phase of the rollout strategy will determine how to get beyond the thought process in the most efficient and effective manner possible, by determining the optimum design for the architecture. Phase 3 will achieve this by helping to ensure that the conceptual straw model technology architecture translates into a pilotable system that has commercial relevance in a highly complex industry, rather than something which is broader than it needs to be or more demanding than target participants will accept. Phase 3 will also ensure that the rollout strategy is wholly objective and not idealistic, which would be an inefficient use of resources and likely discourage adoption.

### Phase 4: Pilot elements of proposed solution

Guided by realistic options developed during the scenario analysis, the 4th phase of the rollout process represents the 1st of a 2‐step pilot process. This phase of the rollout strategy will pilot individual elements or subgroups of the technological requirements to fulfill the capabilities and functions that are proposed for the final technology architecture that will enable interoperable traceability.

### Phase 5: Amalgamation and refinement

Insights and lessons learned during the initial pilots (including refinements to the technology or implementation process) will guide the 5th phase of the rollout strategy. This will see the full system implemented within controllable boundaries to stress test the technologies’ robustness, resilience, and performance in a fully commercial setting. This will be achieved by amalgamating the elements tested separately in Phase 4 into a final technology solution, or series of solutions. To ensure that the system is adequately stress tested prior to rollout, the implementation and evaluation process will include at least 1 supply chain that did not participate in phase 4. With any necessary refinements completed, the technology architecture and enabling solutions will be ready for widespread rollout.

### Phase 6: Full rollout

Supported by education and training efforts, the rollout of the final architecture for enabling interoperable traceability in the seafood industry will occur.

## Leadership Groups Formed

Governance models established to ensure the long‐term success of interoperability solutions in other industries, and implications for the seafood sector, are described in the article “Implementing Interoperability in the Seafood Industry: Learning from Experiences in Other Sectors” in this Supplement. The investigation by Bhatt and others (2017) highlighted the importance of establishing a strong effective governance process from the outset. Having an identifiable, respected, and trusted group of industry leaders at the helm is critical to engendering support for and commitment to processes required to build, implement, and maintain technology solutions and operational processes for enabling successful interoperability. An example is the critical role played by the PTI Leadership Council and associated groups in developing an interoperable traceability system. In a number of ways this group addressed challenges analogous to those facing the seafood industry, especially given the similarities in the 2 industries’ structure and the common avenues (retail and foodservice) through which produce and seafood make their way to consumers.

The process of forming the leadership groups that comprise the governance model would begin by appointing key individuals who:

(1) are represented and recognized by industry as effective, innovative, and influential leaders;
(2) strongly believe in (and have experience of) securing the strategic and operational opportunities that traceability provides individual businesses and overall industry;
(3) possess the expertise required to manage multifaceted projects, budgets, and communications;
(4) have close ties with elements of industry deemed most important to establishing a foundation on which an effective system for enabling global interoperable traceability can be formed; and
(5) will advocate to industry stakeholders for the funding required to develop, test, and implement interoperable traceability solutions, 1st in North America and then internationally.

**Figure 4:**
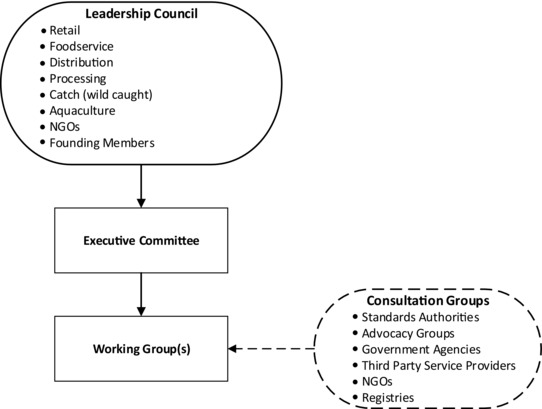
Proposed governance structure.

The proposed governance arrangement shown in Figure 4 reflects findings described in the prior articles contained in this Supplement on interoperable traceability and other research (for example, Sterling and others 2015; Bhatt and others 2016). The Leadership Council and its Executive Committee would provide strategic direction and oversight; the Working Group would have responsibility for implementing the strategy and reporting progress to the Council and funding bodies.

Stakeholder groups identified in Figure 4 are suggestions only. The actual composition (including the number of representatives and their affiliations), along with the invitation and appointment of individuals to each element of the governance structure would occur during the process of finalizing the strategy, which would be achieved through communicating to industry the work achieved to date and capturing feedback. Reflecting learning from an investigation into factors critical to enabling the implementation of effective and efficient interoperability in other industries and sectors (Bhatt and others 2017), each of the 3 groups that together form the proposed governance model would have differing authority and influence over the rollout strategy's finalization and implementation. It is also expected that the governance model would evolve as the initiative matures. Constructive input from wider industry would be assured through the establishment of a 2‐way consultation group that would be consulted at key stages of the rollout process. The Consultation Group could also proactively provide input at any point during the rollout of the technology architecture.

Key functions performed by the governance process would include determining:

(1) who has a say in shaping the initiative's final design and the implementation process;
(2) appropriate funding sources and funding arrangements;
(3) technology decisions—including allowed protocols, standards, and service providers; and
(4) ownership rights and establishment of a sustainable finance model.

## Environmental Scan and Review

The 2nd phase of the rollout strategy is designed to establish the foundational knowledge and capabilities required to enable a diverse set of user needs to be addressed by coordinating and providing support to committed parties. This will assist in fostering the collaboration required to successfully realize the remaining 4 phases of the project. Phase 2 will achieve these objectives by mapping out global trends and determining the extent to which individual seafood businesses are “ahead of the curve” in enabling interoperability and traceability. This will include companies that have built both cutting edge internal traceability systems, as well as companies that have developed or adopted interoperable external traceability systems.

Given the complexity and dimensionality of seafood markets and value chains (Sterling and others 2015; Bhatt and others 2016), the success of the global traceability architecture will rely on managing and embracing this complexity while simultaneously supporting user needs to establish a system that is efficient, cost effective, easy to understand, and user friendly. This will be aided by identifying lessons learned from prior developments of large and complex IT systems that have characteristics similar to those required to meet the seafood industry's needs.

Designing the overarching characteristics of the architecture, including costs, flexibility, and efficiency, will require trade‐off and cost/benefit analysis by project managers in their attempts to optimize the system. Greater speed and efficiency, for example, may significantly increase system costs, resulting in higher customer fees (depending on the business/financial model) but also greater customer satisfaction. Understanding demands of the traceability market and needs of users for architecture attributes will be critical in all phases of rollout design and development. Successfully designing the architecture's “optimal bundle” of attributes, however, will be extremely challenging. The IT “superhighway” is littered with “white elephants” that have failed to meet one or all of their key project objectives (Kozak‐Holland 2007).

It is critical that those participating in the rollout strategy—including the seafood industry, consultants, and IT professionals—have their eyes “wide open” with respect to project requirements, challenges, and opportunities. Phase 2 will therefore review and provide analysis of factors critical to the architecture's successful implementation and sustainability. Biehl (2007) identified that the successful implementation of complex information systems relies on senior management support, well understood business processes, clear project goals, and the management of disaffected employees. Other factors important to success include ensuring a sense of urgency around implementation and having the flexibility to deal with different scenarios. An overall system support person or team is also very valuable.

Seven other factors that will be considered during Phase 2 of the rollout strategy's finalization and planning of the implementation process are:

First, the traceability architecture rollout strategy encompasses both R&D (phases 2 to 5) as well as commercial implementation (phase 6). Project participants must recognize that they are not participating in only a conceptual strategy or limited pilot project, but an all‐out effort to bring to commercial scale and viability the architecture for enabling interoperable seafood traceability. To a major degree that will affect the level of commitment and responsibilities of the players and influence the selection of participants. Scenario development and testing (phase 3), for example, will become a critical exercise, and not simply an abstract game, relative to addressing expected real world needs, issues, and exigencies as well as management and design elements.

Second, extensive research on seafood traceability demonstrates that there is no industry consensus on how businesses operating in the seafood industry should share information or what traceability information should be shared (Sterling and others 2015; Bhatt and others 2016). In larger seafood businesses, there often will not even be consensus among company executives. This lack of consensus reflects the size, diversity, and complexity of the industry, the role of firms within the value chain, the types of products and markets, existence of regulatory mandates, and a host of other factors. In most industries where there has been successful development of traceability systems, the decisions on the “how” and the “what” information is shared are made prior to system build out. The diversity of the industry and lack of consensus will significantly influence the design of solutions to enable the architecture's implementation and drive key needs such as flexibility.

Besides the lack of consensus on “what” and “how” information is shared, the third factor is that there is no consensus on solutions to develop and maintain technology‐enabled interoperability. There is also no agreement on the requirements to support “syntactic” (exchanging information) and “semantic” (interpreting information) interoperability standards and supporting technologies (Sterling and others 2015). Most industries using common traceability and electronic (e)‐information sharing systems have agreements on both syntactic and semantic standards. The rollout strategy must address this issue and recognize that if the architecture is successful, the project will be trailblazing the establishment of global standards for seafood industries and value chains around the globe. This challenge plays out against the larger challenge of the lack of “cross‐domain” interoperability standards among national and international government and fishery management organizations and jurisdictions (NCOIC 2016). Cross‐domain issues are particularly relevant given the high degree of seafood trade across international boundaries as well as the fact that commercial fishery information is often directly managed or significantly influenced by government regulatory regimes. Whether interoperability standards are “open” or “closed” will also be key decisions to be considered during the implementation of the rollout strategy. The utilization of open standards provides valuable benefits throughout the development and implementation of a system, resulting in more sustainable solutions (Bhatt and Gooch 2017; Bhatt and others 2017).

The 4th factor is that there is no agreement on whether interoperability should extend beyond traceability functions, even though doing so could increase the systems’ ability to create value for users (Sterling and others 2015). The earliest requirements for seafood traceability have been driven by government regulatory requirements to address seafood safety, for example the “one up‐one down” traceability system required by the U.S. Food and Drug Administration (Thompson and others 2005). More recently, the United States established a country of origin labeling rule and the Seafood Import Monitoring Program (Blaha 2016) that both require some capacity for supply chain “traceability” consistent with the traceability definition adopted by the GFTC (Borit and Olsen 2012, 1; Olsen and Borit 2013). But neither of these regulations requires e‐interoperability, and information is conveyed using a variety of approaches and technologies. In contrast, sophisticated seafood companies have internal interoperability standards that support a variety of information that can be shared across the company's functions as well as with selected customers on a contractual basis. This information may include methods of production, quality characteristics, and quality control data. Although there may be agreements by individual companies to share information using agreed‐upon interoperability standards and protocols, these agreements are often part of selectively designed contracts that protect intellectual property. Although it may be useful in phase 2 to evaluate examples of these contracts and information sharing that extends beyond typical traceability requirements, a major issue will be determining the extent to which the architecture should support sharing of nontypical traceability information between selected members of the value chain.

Although there has been preliminary discussion regarding required registries to enable the architecture to function efficiently, the fifth factor is that there is no agreement on the registries required to operationalize the architecture, or the required registries’ capabilities. Phase 2 will need to evaluate in in greater detail the types of registries necessary to provide critical categorical information. The capabilities of each registry to enable interoperability and standardization of requisite data will also need to be evaluated. The registries will need to support the categorical information describing the “who,” “what,” “where,” and “when” that enable standardized information to support efficient interoperability across firms and sectors. Registries may include products, premises, participants, movement, and a number of key terminologies. In some cases, participants will need to join a registry and add information; in other cases information will be controlled by the registry manager who will need to periodically update the registries. For these reasons, Phase 2 will need to: (1) review successful (or unsuccessful) examples of registries used in similar global systems; (2) propose which registries to include; (3) determine whether registers already exist and/or could be modified; (4) determine the challenges for developing and using registries; and (5) highlight critical design and management elements. Phases 3 and 4 of the rollout will refine registry selection and design; registries will then be implemented in phases 5 and 6.

A 6th point is that the rollout strategy requires bringing together a strong team of individuals and advisors at key points throughout the project, and always ensuring that individuals’ skills and capabilities are consistent with achieving the goals and needs of each phase. Meeting the traceability needs of diverse clients, including global seafood firms, all industry sectors and value chains, and national or regional governments (where required) will necessitate actively engaged industry champions. Chosen champions will represent a diverse set of firms and sectors, and help achieve full implementation by championing the initiative within their own firm or organization and across the wider industry. The inclusion of technology solution providers at designated points throughout implementation of the technology architectures is also key to success. A team of IT experts that have the expertise required to design P2P architectures and e‐Traceability systems will need to be assembled. Selecting the best champions and IT professionals, and coordinating their engagement throughout the rollout process, will be a critical requirement of project managers.

The 7th factor acknowledges that given the high costs associated with the development and implementation of technology solutions, project systems may initially need to be pilot tested using software‐based simulations. Designing and pilot testing the traceability architecture will be a complex undertaking given the requirements of a global industry comprised of thousands of firms and value chains representing more than 150 countries, producing more than a thousand species/stocks, and generating hundreds of product forms and lot configurations. This will result in literally trillions of combinations of categorical traceability‐related information. A core challenge for the rollout will be designing foundational software systems and pilot testing strategies that encompass critical information needs and system requirements. Given the potentially high cost to develop a pilotable system having the requisite capabilities, the proposed strategy includes using advanced software tools (for example, SPARX Systems Enterprise Architect) to organize and simulate limited scale subsystems in conjunction with scenario analysis and testing (phase 3) and early pilot testing (phase 4).

A framework for transforming distributed P2P capabilities into a form that can be tested virtually prior to commencing the development or modification of software will result from the insights and knowledge produced from having reviewed a variety of environments in which technology and process solutions suited to achieving seafood traceability have been implemented. Incorporating the ability to conduct cost/benefit analysis of potential options will ensure that the framework provides the key knowledge and inputs necessary to accomplishing the next phase of the rollout strategy.

## Scenario Analysis, Design, and Testing

The purpose of phase 3 is to translate the existing conceptual technology architecture and insights that emerge during phase 2 into practical operational interoperable traceability systems whose usability and commercial value will subsequently be pilot tested in phases 4 and 5. This will be achieved by having determined how the technology solutions that result from this phase of the project incorporate the ability to adapt to:

(1) the diversity of needs and complexities inherent to the seafood sector,
(2) the likely adoption pathways utilized by firms and supply chains, and
(3) ensure that the resulting systems are future‐proof.

The adaptability necessary to achieve these outcomes will be built into systems by developing testing scenarios designed to investigate circumstances in which the architecture will operate. The confidence for stakeholders to participate and invest in the implementation of technology solutions will be fostered by having proven the architecture's widespread and persistent commercial relevance in a virtual setting that accurately mimics real‐life situations. This will be achieved by using a combination of scenario analysis and enterprise engineering and software architecture techniques to assess the comparative importance of different functions and capabilities. These functions and capabilities will then be integrated together into the development of robust and resilient interoperable solutions that can evolve with industry needs.

### Scenario analysis

The primary benefit of scenario analysis is that understanding key uncertainties makes design decisions and resulting technology solutions more resilient to changing circumstances. Scenario analysis avoids the stagnation of “wait and see,” by which time the response might be too slow and/or more expensive, as well as the risk of assuming that the future will unfold in a predictable, linear fashion. Scenario analysis complements quantitative, model‐based methodologies by providing the backdrop to recommendations, and prevents judgments against conventional wisdom or spurious expectations of precision. This allows designers and individual firms to assess the capacity of alternative systems to generate return on investment (ROI) in those different contexts. Further, the process identifies the key factors that should be monitored as systems are implemented to inform the nature and timing of subsequent investments.

Examples of considerations that will guide the scenario analysis include:

(1) Can the initial system be ‘basic’ with additional functions added later, and still garner commitment from early adopters?
(2) How important is user friendliness vs. overall value proposition?
(3) What capacity and mechanisms must the design incorporate to secure its initial uptake, and then allow extensibility throughout the adoption pathway?
(4) What are the future requirements and circumstances, especially changes in regulations in different jurisdictions, market demands, and technology, to which the system must adapt and that are critical to delivering the ROI but which are subject to uncertainty in how they will evolve?

The involvement of stakeholders from across value chains operating in developed and developing countries will ensure that the final system is sensitive to commercial considerations that will determine the willingness of managers to adopt the solutions proposed at the conclusion of phase 3. Commercial considerations to foster stakeholders’ commitment, and find partners to pilot the system, include data ownership and the comparative impact of supply chain factors (scale, structure, and state of collaboration) on the ability of stakeholders to benefit from their participation. Determining these critical aspects of the system's design will also inform decisions concerning the appropriate body to lead the initiative, including the role for GFTC given its relationship to industry and respect garnered from a wide range of stakeholders.

### Approach

The scenario analysis will commence with the review of scenario analyses conducted for the seafood sector to determine factors and projected trends identified previously. Three types of scenarios will be developed and tested during this phase of the project. The 3 types are listed below, and described individually.

1) Diversity and complexity scenarios
2) Adoption pathway scenarios
3) Future‐proofing scenarios

Each type of scenario will be developed and tested to expand and challenge current thinking, with the time horizon being the expected lifetime of the architecture being rolled out. The process will include consulting with stakeholders through workshops and interviews. Consulted stakeholders will include key operators from across a variety of seafood supply chains, along with commentators, policymakers, and NGOs. To ensure that the scenarios fully encompass and accurately reflect the realities of North America's seafood industry, including importers, stakeholders consulted will include individuals from a variety of countries. Developing the scenarios will involve provoking people to explore “What if….,” rather than simply stating how they expect the future will turn out. The aim is to emerge with scenarios which are plausible, not probable, thus providing a comprehensive picture of: (1) the circumstances within which firms will decide whether to participate in the architecture, (2) how to make the architecture and resulting solutions suitable for this range of scenarios, and (3) exposing the motivators/deterrents impacting the technology architecture's implementation beyond that which could otherwise occur.

#### Scenario type 1: diversity and complexity scenarios

How does the diversity and complexity of the seafood sector impact on the technological boundaries of what is achievable, and the design of the architecture?

This work stream will involve the development of scenarios that explore the breadth of supply chains in terms of their scale, structure, and capacities, along with the variety of traceability systems for which the architecture needs to be suitable, both initially and eventually. For example, should the technology solutions encompass all seafood species from the outset, or can the system initially focus on only certain species, with extensibilities enabling the addition of species and products without impacting the robustness and resilience of the technologies? The scenarios will also address which capabilities are most vital to enabling the successful implementation of the technologies. For example, given the complexities inherent in the seafood industry and products produced, are scalability (number of dimensions) or extensibility (depth of dimensions) equally important and necessary? Consideration will also be given to the types of governance structures with which the technologies need to comply.

These scenarios will examine whether the solutions need to encompass all or specific drivers of traceability, initially and in the future. Drivers of traceability include food safety, sustainability, combating illegal, unreported, and unregulated (IUU), social issues (fair labor, countering human rights abuse, and so on), fraud, waste reduction efforts, production/marketing efficiencies, and quality control (Sterling and others 2015). Consideration will be given to which of these are likely priorities for early adopters and what succession of additional functions would accelerate the implementation of the architecture and enabling technology solutions?

Insights captured during this analysis will help determine the realistic options and priorities for designing the architecture, and support optimizing the balance of the complexity and capabilities of the technologies. This may include recognition that an aspect of the architecture must be refined ahead of its implementation. Realistic options and any refining of the architecture will depend upon what the scenarios reveal about the key functions, skills, and resources (including financial) required to implement the solutions, to realize an attractive ROI. For example, do considerations such as lifecycle and IUU need to be explicitly addressed? The results will also highlight whether the architecture needs to be applicable to a wide variety of situations from the start, or can be piloted by a similar subset of value chains, and then expanded once it has been validated.

The scenarios will also inform decisions on what organizations are best placed to lead the pilots and subsequently full rollout of the initiative.

#### Scenario type 2: adoption pathway scenarios

**Figure 5:**
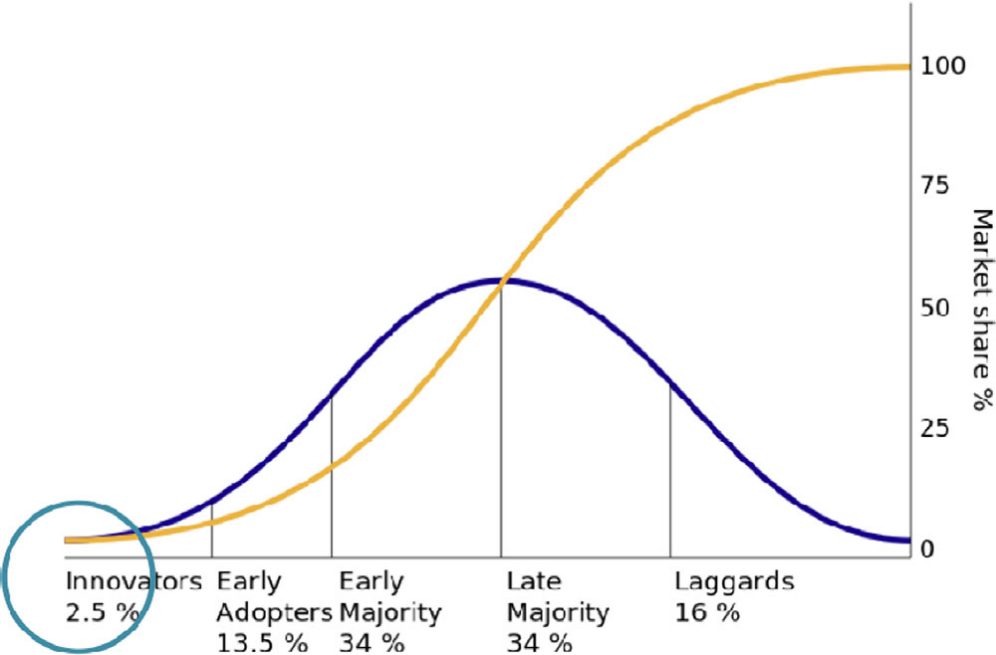
Product adoption curve. Adapted from Rogers (1962). Creative commons, attribution‐ShareAlike 3.0 Unported (cc BY‐SA 3.0), https://creativecommons.org/licenses/by-sa/3.0/.

Rogers (1962) identified that most new technology follows a similar adoption pathway. Accordingly, the 2nd type of scenarios will explore the likely characteristics of firms and value chains in each phase of the pathway presented in Figure 5 and the consequences this has upon the architecture's design and the rollout strategy. The circle overlaid at the 1st stage of the adoption pathway is where Hardt and others (2017) say the seafood industry lies in its use of digital traceability.

Scenarios developed by this workstream would explore the degree to which the type of supply chain in which firms operate impacts management decisions and options available for businesses operating at different points along the supply chain to secure commercial advantage (Gooch and Marenick 2012; Sterling and others 2015). Which of these categories of chains are likely to be early adopters, compared with those that will participate later? The framework developed by the scenario analysis will show the impact of stakeholders’ expectations on how the pilot and subsequent rollout needs to reflect different levels of supply chain collaboration—whether fragmented, cooperative, coordinated, or collaborative.^2^

For example, discussions will ask whether early adopters are more likely to be firms operating in cooperative and collaborative chains. If so, once systems have been validated, should the technologies be adaptable to the needs and barriers associated with coordinated and fragmented chains? Or will fragmented chains never adopt the technologies, unless forced to so, because they are least able to create the value to justify the investment? Conversely, stakeholders operating in collaborative chains may anticipate that they will resolve problems themselves because they trust each other with data and expect to be working together long enough to realize the ROI. If so, then the initial design should target the needs of fragmented and cooperative chains because they are not stable enough to invest in bespoke systems. Therefore, they need a neutral broker for sharing data and a system that they can use with multiple suppliers/customers.

Insights captured from these scenarios will help determine the priorities, technologies, and specifications associated with the initial implementation of the architecture and ensure a rapid rollout. Insights resulting from this 2nd type of scenario analysis will also help determine how and where to implement pilots in the expectation of achieving rapid rollout and highlighting key lessons, and which firms and value chains are likely to commit to participating in these pilots.

#### Scenario type 3: future‐proofing scenarios

The purpose of the 3rd type—future‐proofing scenarios—is to avoid the risk of designing technology solutions that become irrelevant because they cannot adapt to changing circumstances. This scenario analysis will involve identifying the main economic, political/regulatory, technological, market, social, and ecological factors which could influence the architecture's design and implementation. The analysis will then separate identified factors into:

(1) predictable developments—expected to occur over the lifetime of the architecture based on consensus amongst stakeholders;
(2) critical uncertainties—factors whose likelihood, trajectory, or timing cannot be foreseen confidently, therefore making trend extrapolation risky, and/or where responses to a factor's development are unpredictable;
(3) disruptive events—low probability/high impact events to which the architecture must be capable of adapting.

This type of analysis will ensure that the architecture's design encompasses the flexibility to respond to unpredictable or unexpected new requirements or opportunities. This type of analysis prevents decisions from being based on potentially costly assumptions that all identified factors are expected to follow a linear progression of current trends. Instead, the technology designs and rollout strategy produced by this approach will be able to respond at low cost to changing circumstances, so offering a framework for ensuring sustained adoption by having evolved with users’ needs. This will prevent potential participants from being deterred into a “wait and see” attitude.

#### Factors and trends considered

The full range of factors and trends explored in each type of scenario described above will have emerged from the review of existing scenario analyses and subsequent workshops/consultations. Issues likely to be addressed will include consistency in content and timing in the development of regulations in different jurisdictions, such as the U.S.‐proposed IUU rules for imported seafood with traceability requirements that stop at the U.S. importer, and the “one up/one down” traceability required under FDA rules. Incongruences in U.S. and E.U. seafood traceability requirements, or the impact of some nations requiring data to remain housed within their jurisdictions, are other issues that are likely to arise.

The impact of these issues will need to be investigated from the perspective of different governance models, such as the market chain “dominator” model requiring certain information shared all the way through the chain to the “big” retailer, and the impact of different voluntary agreements or contractual arrangements existing among a subset of value chain members on the sharing of information. Issues that might arise surrounding the potential impact of disruptive events might include the collapse of a major global fishery making traceability a much more significant Corporate Social Responsibility issue for retailers and shoppers.

Understanding these types of factors will highlight the critical developments with which the system must be able to cope to ensure its robustness and resilience. The analysis will also highlight the impact of changing circumstances and contextual considerations or trends that might impact implementation and so should be integrated into the design, and monitored from the start. In turn, this analysis will help determine which functions, technology solutions/components, and capabilities are of primary importance, and which could be developed later.

### Virtual testing

A period of virtual testing will see results produced during Phase 2 and the scenario analysis translated into practical technology solution designs that will be pilot tested, 1st virtually and then in practice during phases 4 and 5 of the rollout strategy. Virtual testing, to avoid building a costly “white elephant” system that has limited utility, is gaining increased acceptance as a valuable means to aid the successful transition of conceptual solutions into practical reality. Virtual testing is a particularly valuable approach given characteristics that set the seafood industry apart from a traditional “enterprise.” These include the number, diversity, and complexity of organizations and supply chains that comprise the industry.

As previously described, the process of implementing a technology architecture to enable interoperable whole chain seafood traceability will be extraordinarily complicated. The facts typifying the seafood industry, which create an unprecedented level of complexity compared to other industries in which technology architectures and interoperable solutions have been implemented, include:

(1) hundreds of species, hundreds of product types, and hundreds of lot configurations, each of which may be referred to in different ways by different people;
(2) highly disparate technological capabilities of firms in a supply chain, which range from purely paper‐based data recording and storage to fully integrated Enterprise Management Systems (EMS);
(3) relatively long chains comprised of many different individual firms;
(4) communication occurring in various world languages, even within a single supply chain; and
(5) generation of a vast amount of data that must be collected, stored, analyzed, and shared.

Although no previous examples were found documenting the industry‐wide implementation of a distributed P2P traceability architecture,^3^ a review of literature pertaining to the successful development of complex systems identified the challenge of implementing a technology architecture analogous to the field of enterprise engineering. An enterprise refers to a complex system made up of interdependent pieces (people, information, and technology) that interact with their environment and each other in order to achieve a common goal (Liles and others 1995a,b; Liles and Presley 1996). Although the factors listed above have significant implications for the design and implementation of a global seafood traceability architecture compared to the situations into which other industry‐wide interoperability has been enacted,^4^ many of the lessons learned and tools developed in enterprise engineering are applicable to the seafood industry, especially those pertaining to architectural design and testing.

Enterprise engineering relates to the identification, design, implementation, and continual evolution of enterprises. Although similarities between a single “enterprise” and the seafood industry are apparent from these definitions, enterprise engineering is usually focused on a single organization rather than an industry‐wide scale, with most applications being within a company or corporation. Enterprise modeling explicitly represents both the organizational and technical infrastructure of the system that is being developed (Dietz 2006), and is closely related to enterprise ontology. Enterprise ontology strives to define the “essence” of an enterprise, meaning that it is independent of the current realization of the processes that are undertaken in an enterprise (den Haan 2009). The disciplines of infrastructure and ontology together form the enterprise architecture. A 1st stage in developing an enterprise architecture involves developing a model that identifies enterprise functions, interactions, hardware, and software needs. These models are sometimes referred to as a functional software architecture (Mitra 2008; Microsoft 2016).

The functional software architecture model guides enterprise architects, software developers, and software engineers through the process of developing an integrated and streamlined architecture, by serving as a “big picture” reference (Kosanke and others 1999). These models can be represented using enterprise function diagrams, which represent the different enterprise functions and interactions. In the context of the interoperable seafood traceability architecture, an enterprise function diagram would represent each possible node in the supply chain, the types of data passing through and entering at each node, the possible interactions between each node, and the associated software needs arising from each interaction. The software architecture provides a high level model of a highly complex system by abstracting system components as well as the interactions between components, with tests performed at one stage, then as each additional stage is incorporated, and so on. Called system testing, this technique controls the complexity of the system by conducting a test of each element of the system using an abstract model that is developed in stages (Greis 2013; Liu and others 2015).

The controlled systems approach to developing a functional software architecture informs the design of the enterprise system, including software and hardware requirements to connect computer programs among nodes in the enterprise, or the supply chain in the case of the seafood industry. Enterprise software nodes would be connected through application programming interfaces (APIs) that in totality connect the information system(s) that each firm in the supply chain uses, thereby producing an interoperable network. If designed correctly, it is this series of enterprise software nodes that ensures interoperability (Zelm and Kosanke 2010).

#### Virtual automated testing

There are a number of companies that specialize in testing enterprise systems through automation. Examples of testing tools that are flexible and adaptable to different software methodologies and enterprise systems such as cloud technology include Worksoft and TestPlant. In addition to automated testing, specialized testing is customized to represent the perspectives of enterprise users. An example of specialized testing that is pertinent to the development of the seafood industry is incremental testing wherein each module of the system is tested individually before another module is added in. This, for example, would enable the traceability architecture to be thoroughly tested from the perspective of the 1st node in the value chain before the test is expanded to include more nodes. Specialized testing is an important component of the development and implementation of enterprise systems, often being done while the software and hardware systems are being developed (Myerson 2011).

An added value of virtual testing systems such as SPARX proprietary Enterprise Architect software is that they provide a direct bridge into the practical application of interoperability solutions by enabling automated and specialized usability testing capabilities to be built into enterprise systems during their development and virtual testing. Enterprise architect utilizes simulation of an enterprise, and its interdependencies and information flows to accomplish usability testing in a virtual setting. In other words, the code for the entire system as well as hardware implementations do not need to be written or initialized to ascertain whether a system will work. In addition, different system requirements and designs can be tested to ascertain impacts on system effectiveness and ease of use, thereby enabling virtual testing to seamlessly transition into practical testing of applications and the overall usability of systems following their implementation in real life‐situations. An example of this approach playing an instrumental role in the design, testing, and development of a technology system is American State's health insurance exchange system (Bassett 2013).

## Initial (enterprise level) pilot test

Guided by realistic options developed during the scenario analysis and the design and testing phase, the 4th phase of the rollout process will stress test the robustness and resilience of APIs and associated technologies required to enable interoperable traceability by linking internal and external traceability systems. The document entitled “Specifications to Implement a Technology Architecture for Enabling Interoperable Food Traceability” (Bhatt and Gooch 2017) describes APIs necessary to achieving effective and efficient interoperability. The report presents technology solutions required to enable the operation of secure virtual lock boxes for sharing and accessing data, along with examples of potential vendors.

Similar to the incremental testing of components that will occur in the virtual testing process, the complexity of the rollout will be limited by piloting individual elements of the final system from a one‐up one‐down perspective. The validity of this approach is supported by examples from previous successful initiatives, including the PTI for enabling full‐chain traceability in the U.S. fresh fruit and vegetable industry. The initiative's success is in large part due to their implementation strategy of testing individual components of proposed solutions using actual firms in the supply chain. The direct participation in the pilot process of the individuals who together formed the Leadership Council—which effectively acts as the PTI Board of Directors—ensured that lessons learned at each stage of the system's development were incorporated into the PTI rollout process (Bhatt and others 2017).

Having rigorously tested the proposed solutions virtually and imbedding monitoring capabilities into the technology solutions being tested will further mitigate complexities and risks that can arise in a product introduction of this type. Implementing the pilots alongside current traceability systems will ensure that unintended consequences do not emerge during the pilot that negatively impact firms’ performance. Further justification for testing individual elements of a complex technology in a controlled environment rather than piloting entire systems is provided by Bertolino and others (2003), Biehl (2007), and Liu and others (2015). They cite that this approach reduces the financial and reputational risks and costs associated with a full pilot. This is important given that the majority of seafood consumed in the United States is imported and there are inherent complexities associated with the international seafood industry (Sterling and others 2015; Bhatt and others 2016). Testing the robustness and resilience of a system for enabling chain‐length seafood traceability in a real‐world setting relies on including businesses located not only in the United States but also in other countries and regions of the world. Other reasons for testing specific elements of the final system include that it provides greater opportunity to fully assess the comparative value, cost/benefit, and robustness of each element of the proposed final solution from strategic and operational perspectives.

Testing individual elements of a system also provides a practical means of ensuring the involvement of all necessary stakeholders during the testing and refining of technology capabilities and functions. In line with Biehl's (2007) recommendations on how to successfully implement complex technologies, stakeholder involvement will include the creation of cross‐functional teams that support and champion the pilot in each of the participating businesses. The composition of cross‐functional teams, along with individuals’ roles and responsibilities, will be guided by outcomes produced by the scenario analysis and an explicit acknowledgement that the teams are partnership groups. The purpose of the teams is to support and enable the pilot process, not to form working groups or determine winners and losers.

The 1st phases of the strategy will have determined the businesses, agencies, products and locations suited to each pilot. In addition to the choice of specific technology solutions piloted, the scenario analysis will also have determined the depth and breadth of data used to stress test the chosen systems’ robustness, resilience, and value from functional and commercial perspectives. The scenario analysis will also have determined the initial metrics used to evaluate the performance of systems from functional and commercial value perspectives. Performance metrics will be refined during the pilots, through inputs provided by commercial, government, and NGO stakeholders.

Considerations given towards specific stakeholders participating in the pilots include the need for a mixture of proactive champions and companies highly sensitive about sharing data. The ability to adequately test the system's performance and capacity to meet stakeholders’ needs rests on ensuring that an appropriate cross‐section of industry stakeholders participate in the pilot. Stakeholders will include a mix of commercial businesses and technology providers, possibly along with wider stakeholders such as representatives from NGOs and government agencies. This will ensure that the solutions that result from the pilot are practical and beneficial for businesses operating in supply chains that have different characteristics, while meeting the needs of regulators and aspirations of NGOs.

The way in which the teams interact to establish a continual improvement process and people are motivated to actively engage in the pilots by fostering a sense of urgency will be established at the outset of phase 4. Forming teams to drive continual improvements in the capabilities of technology solutions and encourage the adoption of interoperability reflects the importance that Bates (2014) and Wenger‐Trayner (2015) place on engaging communities of practice to achieve constructive change. Active stakeholder engagement also provides an effective venue for guiding the development of training materials and activities required to enable the wider implementation and adoption of the architecture.

Factors common to all pilots will include the processes used to communicate data, for example open standards; and standardized data gathering, sharing, reporting, and storage protocols; along with common identification protocols. These all relate to the structural principles that are critical to enabling robust resilient interoperable traceability. Reflecting the need for inherent flexibility, the practices used to securely gather, share, transmit, validate, verify, and store data are expected to differ among the participating businesses. The reasons for this include the extraordinary diversity, even within the same supply chain, that exists among harvesters, processors, and suppliers, and the use among most seafood businesses of a combination of electronic and paper‐based processes to manage traceability data (Bhatt and others 2016). The practices employed to gather, record, and share data are factors relating to the operational and integrative principles which, as described by Bhatt and others (2017), are critical to enabling efficient and effective interoperable traceability.

## Amalgamation and Refinement

Phase 5 represents a natural progression in the scale and scope of the implementation of the technology architecture. The knowledge, skills, technology solutions, implementation practices, and materials that result from having piloted individual elements of the system will guide amalgamation of these elements into an end‐to‐end technology solution, resulting in interoperable chain‐length traceability. To reduce the risk of unanticipated situations or occurrences undermining the testing and evaluation process, the pilot will be bounded within specific species, products, participating businesses, and clearly‐defined supply chains. The testing process will be strengthened by extending the pilot beyond businesses and stakeholders who participated in the previous phase of the rollout.

Delivered along similar lines to the initial pilot process described previously, 3 primary purposes lie behind this phase of the rollout strategy. The 1st purpose is to stress test the systems’ robustness, resilience, performance, functions, and capabilities when implemented in multiple supply chains stretching from catch to consumer. The 2nd primary purpose is to validate the query and response mechanisms prior to rolling out the technology architecture and enabling solutions to the wider industry. Third, this phase of the rollout strategy will evaluate the effectiveness of processes designed to support the technology architecture's implementation in a range of locations and among businesses having differing technical or management capabilities.

Activities performed during this phase of the rollout will include delivering preparedness and support training throughout the implementation of the system. Testing of the system's robustness and integrity will include conducting mock recalls. Data and process audits will also be conducted on multiple occasions at different points along the supply chain. Technology support, combined with the mirroring capabilities imbedded in the technology solutions during Phase 3, will ensure that elements tested separately in the previous stage of the rollout strategy interact as designed and do not negatively impact the legacy systems of participating businesses.

At the conclusion of Phase 5, the resources required to implement the interoperability‐enabling solutions in environments foreseen during the 1st phases of the rollout, will have been established. These resources will include technological capabilities and expertise, awareness and training materials, and outreach activities. The nature of specific support and enabling capabilities will differ according to whether they are delivered to commercial enterprises, third‐party solution providers, IT consultants, and noncommercial stakeholders, such as governments or NGOs.

## Full Rollout

The final phase of the rollout strategy will see the technology architecture and enabling solutions promoted widely to the seafood industry. During prior phases of the rollout strategy, it will have been determined how performance of the technology architecture and enabling solutions will be monitored and reported to industry stakeholders, for the purpose of increasing the breadth of adoption and enhancing the value of the architecture to industry.

The extent to which the full rollout is staged by incrementally targeting individual sectors and jurisdictions according to certain species, products, or other criteria will have been determined in prior phases. The timing of the full rollout will also depend on lessons learned and capabilities developed during prior phases, along with the location and availability of resources required to implement the technology architecture on a broad scale. Required resources include the funding of support materials and industry outreach. Constraints that may impact the timing and scale of the full rollout include technology related infrastructure, businesses’ internal capabilities and, potentially, government policies, legislation, and regulations.

Successful implementation of the technology architecture will require that the oversight process established at the outset of the implementation of the strategy evolve into a self‐sustaining governance and finance model.

## Conclusions, Next Steps

We proposed a rollout strategy for implementing a technology architecture designed to enable interoperable traceability in the global seafood industry. As described in other articles in this Supplement (Bhatt and others 2017; Hardt and others 2017; Lewis and Boyle 2017) and other research (for example, Bhatt and others 2016; Bhatt and Gooch 2017), interoperable traceability offers opportunities for businesses operating in the seafood industry to improve their performance and strengthen their competitive advantage. Interoperable traceability also offers the opportunity to improve the seafood industry's environmental sustainability and achieve societal benefits that would likely not otherwise be possible. We proposed a means of achieving these outcomes in an effective and efficient manner, by coordinating existing capabilities and technical solutions in one overarching strategic initiative. The greatest challenge that the strategy may face is addressing the attitudes and behaviors that so far have limited the adoption of seafood traceability.

Next steps for enabling the implementation of the proposed strategy are finalizing its design, which will include establishing realistic timelines for each phase of the work, forming a governance structure (recognizing that governance will evolve as the initiative progresses towards to the full rollout phase), and securing the resources required to embark on implementing the strategy. This process will include the following:

(1) Communicating the draft rollout strategy and the opportunities it presents to industry.
(2) Engaging industry in formalizing the strategy and establishing a leadership team.
(3) Estimating the costs and resources required to implement each phase of the strategy.
(4) Securing the resources required to begin the implementation process.
(5) Initiating the strategy, beginning with the proposed environmental and literature review.

## Footnotes

1 “The ability to access any or all information relating to that which is under consideration, throughout its entire life cycle, by means of recorded identifications.”

2 For a description of characteristics pertaining to the 4 types of supply/value chain and the use of traceability for commercial purposes, see Project to Develop and Interoperable Seafood Traceability Technology Architecture: Issues Brief (Bhatt and others 2016).

3 The closest example identified is California's E‐Pedigree traceability system for pharmaceutical products.

4 Examples of implementation of industry‐wide interoperable traceability include the finance, fresh produce, travel, and automotive industries.
